# Phylogeographic and genome-wide investigations of Vietnam ethnic groups reveal signatures of complex historical demographic movements

**DOI:** 10.1038/s41598-017-12813-6

**Published:** 2017-10-03

**Authors:** S. Pischedda, R. Barral-Arca, A. Gómez-Carballa, J. Pardo-Seco, M. L. Catelli, V. Álvarez-Iglesias, J. M. Cárdenas, N. D. Nguyen, H. H. Ha, A. T. Le, F. Martinón-Torres, C. Vullo, A. Salas

**Affiliations:** 1 Unidade de Xenética, Departamento de Anatomía Patolóxica e Ciencias Forenses, Instituto de Ciencias Forenses, Facultade de Medicina, Universidade de Santiago de Compostela, Galicia, Spain; 2 GenPoB Research Group, Instituto de Investigaciones Sanitarias (IDIS), Hospital Clínico Universitario de Santiago, Galicia, Spain; 3 Translational Pediatrics and Infectious Diseases, Hospital Clínico Universitario de Santiago, Santiago de Compostela, Spain; 4 GENVIP Research Group, Instituto de Investigación Sanitaria de Santiago, Galicia, Spain; 5 Equipo Argentino de Antropología Forense, Independencia, 644 Córdoba Argentina; 6 Grupo de Investigación en Genética Forense – Instituto Nacional de Medicina Legal y Ciencias Forenses, Bogotá, Colombia; 7 National Institute of Forensic Medicine, Ministry of Health, Ha Noi, Vietnam

## Abstract

The territory of present-day Vietnam was the cradle of one of the world’s earliest civilizations, and one of the first world regions to develop agriculture. We analyzed the mitochondrial DNA (mtDNA) complete control region of six ethnic groups and the mitogenomes from Vietnamese in The 1000 Genomes Project (1000G). Genome-wide data from 1000G (~55k SNPs) were also investigated to explore different demographic scenarios. All Vietnamese carry South East Asian (SEA) haplotypes, which show a moderate geographic and ethnic stratification, with the Mong constituting the most distinctive group. Two new mtDNA clades (M7b1a1f1 and F1f1) point to historical gene flow between the Vietnamese and other neighboring countries. Bayesian-based inferences indicate a time-deep and continuous population growth of Vietnamese, although with some exceptions. The dramatic population decrease experienced by the Cham 700 years ago (ya) fits well with the Nam tiến (“southern expansion”) southwards from their original heartland in the Red River Delta. Autosomal SNPs consistently point to important historical gene flow within mainland SEA, and add support to a main admixture event occurring between Chinese and a southern Asian ancestral composite (mainly represented by the Malay). This admixture event occurred ~800 ya, again coinciding with the Nam tiến.

## Introduction

The Republic of Vietnam, located in the easternmost end of the Indochinese Peninsula (South East Asia; SEA), is a country conformed by a rugged and mountainous strip of land with many coastal plains and deltas. It borders China and the gulf of Tonkin in the North, Laos and Cambodia in the West, and the gulf of Thailand in the South^1^. It is one of the most populous countries in the world; according to estimates by the Vietnam General Statistics Office, in 2016 more than 94 million people lived in the country (Vietnam General Statistics Office [VGSO]: www.gso.gov.vn; accessed March 2017). Hanoi, the capital of the country, is located in the northern region and has around seven million inhabitants; it is the second most populated city in Vietnam after Ho Chi Minh (old Saigon), located in the southeastern most part of the country (>8.4million of inhabitants).

Different populations and ethnic groups have influenced present-day Vietnamese communities. Thus, Vietnam is today a multi-ethnic country; its government recognizes 54 ethnic groups, of which the Kinh account for the vast majority of the country (87%; ~77 million people) and are widespread across the whole territory. The remaining 13% are divided into 53 other groups, officially recognized as ethnic minorities, that are dispersed over hilly areas (highlands cover three fourths of the Vietnamese territory) spreading from the North to the South. Among the minorities, the largest is the Tay group, conformed by 1.7 million people, followed by the Khmer Thai, Hoa, Mong, and Nung ethnic groups with a population of roughly one million each; the least populous are the Brau, Roman and Odu, conformed by only a few hundred people each^1^, VGSO).

South Asia (SA) was one of the first regions to have been peopled by modern humans; and this region has served as a major route of dispersal to other geographic areas, including SEA^2^. According to Atkinson *et al*.^3^, roughly 60% of the global human population lived in SEA about 38,000 years ago; and Vietnam was one of the first world regions practicing rice agriculture^4^. Two main hypotheses have been proposed to explain the initial geographic/population sources contributing to present day SEA populations. The first one proposes that populations to the South of East Asia (EA) probably derived from the populations in SEA that migrated from Africa, possibly *via* mid-Asia following a coastal route^5^. The other hypothesis proposes at least two independent migrations: the same initial movement coming from Africa following a southern coastal route first, followed by a series of migrations along a more northern route that served to bridge European and EA populations^6^. According to the latter hypothesis, most Vietnamese ethnic groups today would be descendants from the ancient populations that spread from South of the Yangtze River towards Mainland SEA and the SEA islands^1^. However, numerous migrations and successive integration processes could have occurred over time, modeling the genetic composition of SEA^5^. For instance, Vietnam has been also involved in important commercial historical routes, such as the Silk Roads, and since more than 2,000 years ago, the Vietnamese coast has attracted merchants from as far away as the Middle East and Japan. These commercial exchanges have contributed much to the cultural^7^ and genetic exchange among Eurasian populations.

In recent years, SEA has been extensively explored in genetic studies. Some mitochondrial DNA (mtDNA) studies have suggested that this region constituted the starting point of the modern human expansions from Africa towards China, EA and Oceania^8,9^. The remarkable (pre)-historical population growth of this region has paved the ground for the incubation of high genetic diversity in this area. Thus, SEA’s main mtDNA haplogroups (M, B, R and F) appeared over 50,000 years ago^10^, and they show a remarkable genetic variability within clades. Soares *et al*.^11^ suggested that global warming and sea-level rises at the end of the Ice Age, were the main forces driving human diversity in SEA.

Despite the numerous studies carried out so far, knowledge about the mtDNA variability of many SEA countries such as Vietnam, Burma, or Cambodia is still very limited^9,12^. One of the first studies aimed at unraveling the mtDNA composition of SEA countries was conducted by Oota and colleagues^13^. They analyzed a Vietnamese sample set collected from first generation South Vietnamese immigrants from California and compared it with sample sets collected from Han Chinese and Japanese. Their results showed that the Vietnamese sample had the highest levels of within-population mtDNA variation. Li and colleagues^8^ analyzed a Vietnamese and a Chinese sample set, and reported high frequency of haplogroups B, M7, F and R, which are common in SEA and key to understand the history of SEA populations. Irwin and collaborators^14^ analyzed a large sample set of Vietnamese mtDNA control region (CR) sequences with a predominantly forensic genetic focus. Their data indicated that the northern Vietnamese population has a high genetic diversity. Zimmerman *et al*.^15^ described high haplotype diversity in northern Thailand and reported that 14% of the Thai haplotypes were shared with other SEA populations. Bodner *et al*.^12^ analyzed the mtDNA composition of Laos, indicating that this population showed a mtDNA diversity pattern characteristic of SEA, where B4a, B5a, M7b1, F1a and R9 (the latter two referred in this article as R9’F) are the most frequent haplogroups. They also reported that many of the haplotypes found in the sample were shared with surrounding populations, mainly Thailand and Vietnam^12^. Zhang and colleagues^16^ analyzed an extensive mtDNA sample of aboriginal Cambodians and sequenced 98 mitogenomes; these authors suggest that Cambodian aborigines are descendants from ancient populations, and their results add support to the theory that modern humans initially settled in SEA and then dispersed towards China and the SEA islands. According to these authors, Cambodia might be the center of dispersal of B5a, F1a, M12b and B4c2 haplogroups to northern regions and mainland EA^16^. More recently, Summerer and collaborators^9^ investigated a Myanmar population sample set and sequenced 44 mitogenomes. They concluded that Myanmar displays a distinctive SEA haplogroup composition, but with northeast Asian and Indian influences. Their analyses indicated that the migration rates between Myanmar and Vietnam were approximately equal^9^.

A large number of studies have pointed to SEA as the cradle of present-day Asian populations^3^. An important effort at characterizing the SEA area in terms of mtDNA variability has been carried out in the last decades^9,12,15,16^. The present project aims at contributing to improve our knowledge about the genetic variation of this region, given the importance of this region for the dispersion of modern humans in Asia. Although a few mtDNA studies have been conducted so far, the mtDNA data available from Vietnam is still scarce. To the best of our knowledge, the present study represents the largest sampling effort in Vietnam to date. A genome-wide analysis of a Vietnamese sample was also carried out to further test hypotheses suggested by the mtDNA data.

## Material and Methods

### Sample collection

A total of 622 Vietnamese samples were recruited from six different locations: Cao Bang (*n* = 113), Lao Cai (*n* = 115), Hanoi (*n* = 38), Hai Phong (*n* = 133), Da Nang (*n* = 135), and Ho Chi Minh (*n* = 88). For some analyses, the samples were collapsed into three geographic regions: North (*n* = 399), Center (*n* = 135) and South (*n* = 88). The samples were also analyzed according to their self-reported ethnic group adscription: Tay (*n* = 62), Kinh (*n* = 399), Hmong or Mong (*n* = 115), Hoa (*n* = 23), Nung (*n* = 21), Kho Me (*n* = 1) and Thai (*n* = 1). Table S1 shows detailed information about sampling location, birthplace, and ethnic group. An additional 724 samples from Vietnamese were collected from the literature for comparison^8,13,14,17,18^. Our newly generated data cover regions and ethnicities from Vietnam hitherto unexplored in the literature, such as Lao Cai, Da Nang and Cao bang, as well as some ethnic minorities (e.g. Mong, Tay, Nung…).

We obtained written informed consent for all donors prior the research, which includes consent for publication of individual data. The rights of participants were safeguarded during the research and their identity was protected. The experimental protocol was approved by the ethical committee of the National Institute of Forensic Medicine (Ministry of Health, Hanoi, Vietnam). Moreover, the study conforms to all applicable Spanish normative, namely the Biomedical Research Act (14/2007-3 of July), the Autonomy of the Patient Act (41/2002), Decree SAS/3470/2009 for observational studies and the Data Protection Act (15/1999).

### PCR and mtDNA sequencing

mtDNA CR comprising hypervariable regions (HVS-I/II) were amplified using PCR primers previously reported^19^. PCR was carried out in a 10 µL final reaction volume consisting of 3 µL QIAGEN master mix, 1 µL for each PCR primer (5 µM), and 2 µL of DNA (5 ng). Amplification was carried out in a GeneAmp PCR System 9700 (Applied Biosystems, Foster City, California, USA) using a hot start at 95 °C for 15 min and followed by 30 cycles at 94 °C for 30 s, 58 °C for 90 s, 72 °C for 90 s and a final extension at 72 °C for 10 min.

Prior to the sequencing reaction, PCR products were checked by electrophoresis in polyacrylamide non-denaturing gel (T9, C5), and subsequently stained with silver nitrate. PCR products were then purified with a Multiscreen PCR_µ96_ Plate 96-well device (Millipore, Bedford, MA 01730, USA).

The Sequencing reaction was performed on both strands (when needed) in a GeneAmp PCR System 9700 (AB) using the BigDye® Terminator v3.1 Cycle Sequencing Kit (AB). The sequencing reaction was carried out using 0.5 µL of BigDye Terminator Kit, 2 µL of BigDye terminator Buffer, 1 µL PCR Forward or Reverse primer, 3.3 µL of PCR purified product in a final volume of 10 µL. The sequencing program consisted of a hot start at 96 °C for 3 min, followed by 25 cycles at 96 °C for 30 s, 50 °C for 10 s, and 60 °C for 4 min. Sequencing products were purified using EDGEBIO purification plates (Perfoma®V3 96-Well Shortplate), following the manufacturer protocol; capillary electrophoresis was done in ABI PRISM 3730 Genetic Analyzer (AB).

The resulting data were analyzed with SeqScape Software 2.1 (AB) from position 16024 to16569 for HVS-I and from position 1 to 576 for HVS-II.

### Complete genomes

With the aim of investigating in detail the phylogenetic characteristics of some of the mtDNA sequence motifs observed in our Vietnam samples, we additionally investigated the mitogenomes provided by The 1000 Genomes Project (1000G), and the literature. The 1000G dataset consists of 101 samples collected from Kinh donors from Ho Chi Minh City. The data was processed as reported before^20^. New haplogroup labels were assigned to new phylogenetic branches when suggested by at least two mitogenomes, and when additionally supported by CR data; e.g. ref.^21^. In order to further investigate the phylogeny of haplogroups F1f and M7b1a1f, we further collected mitogenomes from the literature and GenBank belonging to these clades (*n* = 129). The mitogenomes used in the present study were compiled in Table S2.

### Statistical and phylogenetic analysis

Diversity indices were computed on mtDNA sequences using DnaSP v.5 with the complete CR (16024–576) range^22^. Indels were excluded from these calculations. Genetic structure and variation between individual and grouped population sets were carried out by means of Analysis of Molecular Variance (AMOVA) and *R*_*ST*_ genetic distances as implemented in Arlequin software v3.5.1.2, and using the CR sequences^23^.

Spatial geographic representation of haplogroup frequencies was carried out using R Project for Statistical Computing v. 3.3.1 (https://www.r-project.org/). We followed the commonly used ordinary Kriging method, implemented in the automatic interpolation package *autoMap* v. 1.0-14 (https://cran.r-project.org/web/packages/automap/index.html), for interpolating frequency values (other interpolated methods yielded virtually the same results).

Mitochondrial DNA haplogroup inference was done using Haplogrep2^24^. Haplogroup classification was manually checked and following PhyloTree Build 17 (18 February 2016)^25^. Principal Component Analysis (PCA) was carried out with the haplogroup frequencies reported by Zhang *et al*.^16^ on 180 SEA populations, and using R software.

The software popART (http://popart.otago.ac.nz) was used to generate phylogenetic networks of mtDNA haplotypes using the median-joining algorithm^26^, and considering the HVS-I region from position 16051 to 16400.

### Time to the most recent common ancestor

The time to the most recent common ancestor (TMRCA) for the phylogenies generated in the present study was estimated using the maximum likelihood (ML) procedure implemented in PAML 3.13^27^ and assuming the HKY85 mutation model, gamma-distributed rates (approximated by a discrete distribution with 32 categories), and three sequence partitions (as in e.g. ref.^28^). Hotspot mutations were excluded from the calculations. Mutational distances were converted into years using the corrected evolutionary rate proposed by Soares *et al*.^29^ and a generation time of 25 years.

### Effective population growth through time

Extended Bayesian Skyline Plots (EBSPs)^30^ were obtained using BEAST v1.845 for the CR sequences and mitogenomes analyzed in the present study as done previously^28^, in order to infer effective population size (*N*_e_) through time and demographic changes from the data. We estimated the best fitting substitution model from our data using jModeltest^31^. The 1000 G F1f (*n* = 97) and M7b1a1f (*n* = 32) mitogenomes were aligned and divided in two partitions: the coding (positions 577 to 16023) and non-coding region (positions 16024 to 576). We used the mutation rate of 9.883 × 10^−8^ substitutions/nucleotide/year for the non-coding partition, and 1.708 × 10^−8^ substitutions/nucleotide/year for the coding region^32^. Three independent Markov chain-Monte Carlo runs of 50,000,000 steps each were performed, with samples taken every 1,000 steps. After inspecting Tracer (v1.6) outputs for distribution convergence, the runs were combined using LogCombiner v1.8.2, with 10% discarded as burn-in, for a final 135,000,000 total steps. The csv file from the combined runs was generated through the VDAanalysis/CSVexport functions in BEAST and plotted using R software. EBSP for the main ethnic groups was generated using the control region fragment 16051–16400 from all available sequences and a mutation rate of 1.62 × 10^−7^ ^32^. Three independent Markov chain-Monte Carlo runs of 100,000,000 steps each were performed and subsequently combined. Demographic plots were obtained using the Java scripts from O’Fallon and Fehren-Schmitz^33^.

### Inter and intra population gene flow analysis

We used MIGRATE-n software v3.6.11^34^ to study inter and intra population gene flow in Vietnamese population. Mitochondrial DNA HVS-I segment from 16051 to 16400 was extracted from each population sample, and the ts/tv ratio and gamma shape parameter of the datasets were estimated with MEGA7 software^35^ through four gamma categories and HKY substitution model (most similar to the F84 model used by MIGRATE-*n*). We used all available sequences from Vietnam. We employed a Bayesian approach, which has been demonstrated to be more efficient to obtain reliable results with less effort than the ML procedure when using a single locus^36^. Firstly, we estimated the migration rate between the main language groups in Vietnam: Austroasiatic (Vietic-Kihn, Hre, Mnong, Trieng), Tai-Kadai (Nung, Tay, Thai), Hmong-Mien (Hmong) and Cham (the Chinese group represented by Hoa people was disregarded due to its low sample size). We compared the full migration model, which allows migration between all sub-population groups, to the panmictic model, which assumes that all subpopulations are very similar to each other due to a large gene flow between them through time. Subsequently, we studied the genetic exchange between the Kinh (which represent 86% of the population of Vietnam) and two available populations sets from neighboring countries, namely Cambodia (*n* = 1054) ^16^ and Laos (*n* = 214)^12^. We compared three different models with the population pairs Cambodia-Vietnam and Laos-Vietnam: the full migration model, the unidirectional migration models, and the panmictic model. For this purpose we calculated the model probability and log Bayes factor (LBF) of each model through the log marginal values using the thermodynamic integration method^37^. The best model will give the highest log mL and the model ranking is made comparing the LBF’s and probabilities with respect to the best model.

$$\begin{array}{rcl}{\rm{LBF}} & = & 2(\mathrm{ln}({\rm{mL}}({{\rm{model}}}_{1}))-\,\mathrm{ln}({\rm{mL}}({{\rm{model}}}_{2})))\\ {\rm{Prob}}({{\rm{model}}}_{{\rm{i}}}) & = & \frac{{{\rm{mL}}}_{{{\rm{model}}}_{{\rm{i}}}}}{{\sum }_{{\rm{j}}}^{{\rm{n}}}{{\rm{mL}}}_{{{\rm{model}}}_{{\rm{j}}}}}\end{array}$$

For the best model, we calculated two parameters: the mutation-scaled effective immigration rate (M) and the mutation-scaled effective population size (*Theta*). The parameter M expresses how much more important immigration is for the population compared to mutation. Additionally, we calculated the number of immigrants (*N*_*m*_) in the receiving population as follows: x*N*_*m*_ = *Theta* * M. For mtDNA data and assuming a sex ratio of 1:1 we can consider that the inheritance scalar x equals 1 (Table S4). The static heating option with the standard four chains, interval one and the heating scheme suggested by default was employed. We used 100 randomly picked individuals from the compared populations to avoid potential problems derived from the large as well as the uneven sample size of the datasets. The length of the MCMC chain and the range of the M and *Theta* priors were adjusted until the convergence was reached for all parameters and no priors boundaries problems were detected. We carried out 10 replicates per run. We finally checked the convergence of the runs using *mtraceR* package for R software^38^, which aims to facilitate the diagnostics of Bayes MCMC analysis run with Migrate-n.

### Genome-wide analyses

SNPs from two main genome datasets^39,40^ were intersected, and a total set of 54,780 SNPs were used for analyses. These two datasets represent a large amount of Asian populations; however, since the present study focuses on the Vietnamese only, we reduced the number of population samples used (Table S3). The preparation of the data for analyses was done as described previously^41^. We first computed identity-by-state (IBS) values from the data using PLINK^42^. Multidimensional scaling (MDS) carried out on IBS values were built to discriminate clusters of genomic variation and employing *cmdscale* (library *stats*) from R software.

We then used ADMIXTURE software to estimate maximum likelihoods of individual ancestries from multi-locus data, considering populations from mainland SEA, neighboring populations, and a further two reference populations, namely Europeans (CEU), and sub-Saharan Africans (YRI).

Computation of *f3*-statistics was carried out by measuring allele frequency correlations between populations^43^. The *f3*-statistic values were obtained using *f3* (CHS,Y;KHV); which formally test whether a target population (KHV; Vietnamese) is admixed between the two source populations, namely CHS (southern Han Chinese population) as the main representative of the EA component and different populations from SEA (Y). We also computed the four-population *D-*statistics^44^ to formally test for admixture between Chinese and Malayans, given that these populations appear to be the best subrogates of the populations that contributed to the present pool of Vietnamese, as suggested by the other analyses. Given that this test provides statistical evidence of the direction of the gene flow, *D*-statistics were built as *D*(CHS, KHV; Y, OUTGROUP) and as *D*(Y, KHV; CHS, OUTGROUP), which test for the significance of the contribution of different SEA populations (Y) to KHV and the significance of the CHS contribution under different SEA scenarios (Y), respectively. The weighted block jackknife procedure was used for the computation of *D*-statistics, using a block size of 5MB. The OUTGROUP was built using ancestral alleles at all sites of the genome^45^. Values below -2 are considered to be statistically significant.

ALDER^46^ was finally used to date admixture events in present-day Vietnamese, using the one-reference-population model.

## Results

### Molecular diversity

**Figure 1:**
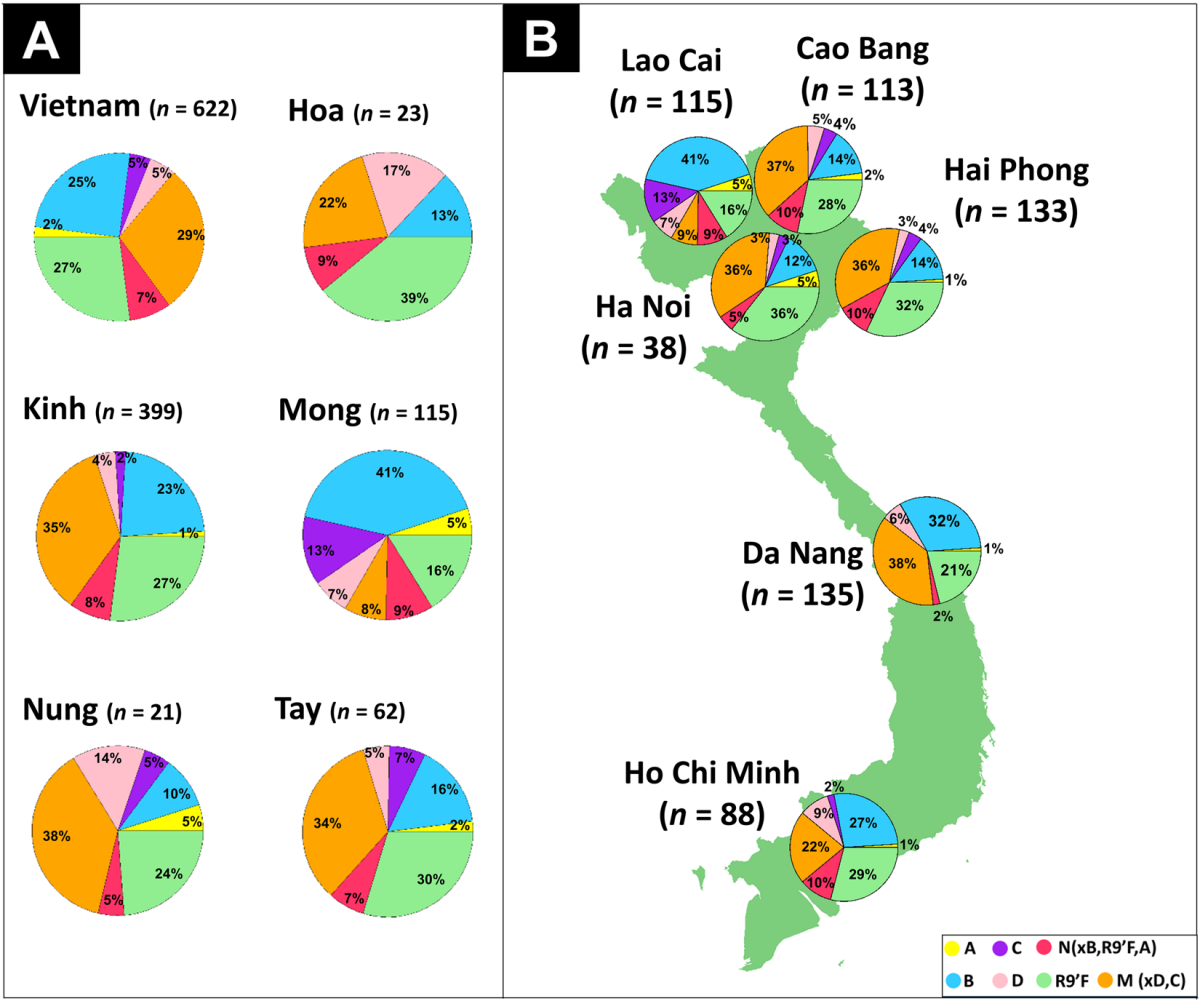
(**A**) Frequencies of main haplogroup and sub-haplogroups by ethnic groups. (**B**) Map showing the location of the main Vietnamese regions analyzed in the present study. The pie charts display the frequency values for the main haplogroup categories. Maps were generated using R Project for Statistical Computing v. 3.3.1 (https://www.r-project.org/) and the package *autoMap* v. 1.0–14 (https://cran.r-project.org/web/packages/automap/index.html). Packages *sp* v. 1.2–5, *rgdal* v. 1.2–8, *gstat* v. 1.1–5, *raster* v. 2.5–8 and latticeExtra v. 0.6–28 were also used to improve visual appearance of the maps.

The present study included 622 DNA samples sequenced for the CR, from six different Vietnam locations that represent seven ethnic groups. There are 476 different haplotypes among Vietnamese mtDNAs, which is testament to a large mtDNA diversity. For the seven ethnic groups represented in our data, only five of them have sample sizes that allow estimating diversity values (Kho Me and Thai were excluded from these computations). Molecular diversity indices in all locations and population samples analyzed were high for both haplotype (*H*) and nucleotide diversity (*π*) (Table 1). The lowest values of haplotype diversity (*H* = 0.980) and nucleotide diversity (*π* = 0.0089) were found in the Mong population. Our Mong sample lived in Lao Cai province **(**Table 1; Fig. 1B), and they represent the second most frequent ethnicity in this province ^47^. At the opposite end of the spectrum are the Hoa, which showed the highest diversity values (*H* = 1.000; *π* = 0.0107). Although the sample of Ha Noi is small (*n* = 38), it is worth highlighting the high values of diversity observed (*H* = 1.000; *π* = 0.0110) (Table 1).

**Table 1:** Summary statistics of CR sequences in Vietnam carried out on six Vietnamese locations. All the computations were undertaken on the common sequence segment that ranges from 16024 to 574. Main regions represent the following populations: North = Lao Cai + Cao Bang + Ha Noi + Hai Pong, Center = Da Nang, and South = Ho Chi Minh.

Population	n	k	k/n	S	n_mut_	H ± SE	Π ± SE	M
Provinces								
Cao Bang	113	108	0.96	147	151	0.999 ± 0.001	0.0103 ± 0.0004	11.5
Hai Pong	133	119	0.89	150	156	0.997 ± 0.001	0.0105 ± 0.0003	11.7
Ha Noi City	38	38	1.00	89	91	1.000 ± 0.006	0.0110 ± 0.0005	12.3
Lao Cai	115	65	0.57	88	91	0.980 ± 0.005	0.0089 ± 0.0003	9.9
Da Nang	135	123	0.91	148	153	0.998 ± 0.001	0.0102 ± 0.0002	11.4
Ho Chi Minh	88	83	0.94	133	137	0.998 ± 0.002	0.0109 ± 0.0004	12.1
Main regions								
North	399	309	0.77	216	230	0.997 ± 0.001	0.0104 ± 0.0002	11.6
Center	135	123	0.91	148	153	0.998 ± 0.001	0.0102 ± 0.0002	11.4
South	88	83	0.94	133	137	0.998 ± 0.002	0.0109 ± 0.0004	12.1
Ethnic group								
Hoa	23	23	1	70	71	1.000 ± 0.013	0.0107 ± 0.0007	12.0
Kho Me	1	—	—	—	—	—	—	—
Kinh	399	334	0.84	218	230	0.998 ± 0.000	0.0105 ± 0.0002	11.7
Mong	115	65	0.57	88	91	0.980 ± 0.005	0.0089 ± 0.0003	9.9
Nung	21	21	1	54	54	1.000 ± 0.015	0.0094 ± 0.0006	10.5
Tay	62	54	0.87	114	118	0.998 ± 0.003	0.0110 ± 0.0005	12.2
Thai	1	—	—	—	—	—	—	—
*Vietnam (all)*	622	478	0.77	252	271	0.998 ± 0.000	0.0105 ± 0.0001	11.7

*n* = Sample size; *k* = Number of different haplotypes; *S* = number of polymorphic (segregating sites); *n*_mut_ = total number of mutations; *H* = haplotype diversity and standard error; *π* = nucleotide diversity and standard error; *M* = average number of nucleotide differences.

**Figure 2:**
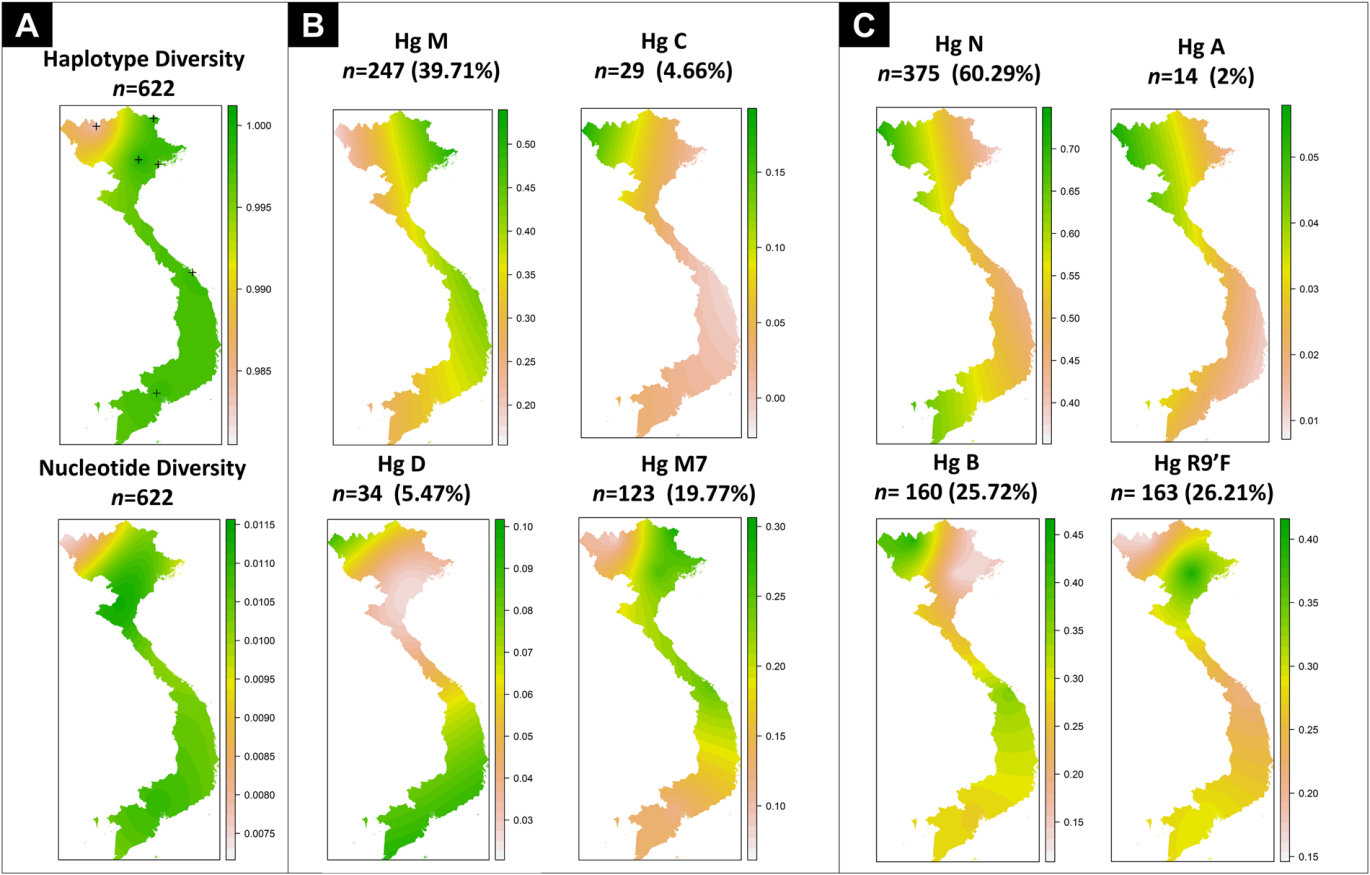
(**A**) Interpolated geographic maps of haplotype diversity (crosses indicate sample points) and nucleotide diversity values. (**B**) and (**C**) Interpolated maps of main haplogroup frequencies across the territory of Vietnam. Maps were created as in Fig. 1.

There is no obvious correlation between sampling locations and molecular diversity as measured by statistical summary indices. However, when the samples were analyzed attending to main geographic regions (North, Center and South), a clearer pattern of molecular diversity is revealed, suggesting that the diversity increases from North to South of the country (Table 1; Fig. 2A).

### Haplogroup patterns and phylogeographic features of Vietnamese haplotypes

Typical SEA haplotypes predominate in the Vietnamese, mainly represented by haplogroups M (39%; with sub-haplogroup M7 [20%] being the most frequent sub-lineage), and haplogroup N (61%; with haplogroup R9’F [27%] and haplogroup B [25%] as the most frequency sub-lineages within N) (Table 2, Fig. 1).

**Table 2:** Haplogroup frequencies in Vietnam by sample location and ethnic groups.

Sample origin	*n*	M	C	D	M7	M(×D,C)	N	A	B	R9’F	N(×A, B, R9’F)
Sample locations											
**Cao_Bang**	113	0.46	0.04	0.05	0.29	0.37	0.54	0.02	0.14	0.28	0.10
**Da_Nang**	135	0.44	0.00	0.06	0.25	0.38	0.56	0.01	0.32	0.21	0.02
**Ha_Noi_City**	38	0.42	0.03	0.03	0.26	0.36	0.58	0.05	0.12	0.36	0.05
**Hai_Phong**	133	0.43	0.04	0.03	0.23	0.36	0.57	0.01	0.14	0.32	0.10
**Ho_Chi_Minh**	88	0.33	0.02	0.09	0.12	0.22	0.67	0.01	0.27	0.29	0.10
**Lao_Cai**	115	0.29	0.13	0.07	0.04	0.09	0.71	0.05	0.41	0.16	0.09
**Vietnam (all)**	622	0.39	0.05	0.05	0.20	0.29	0.61	0.02	0.25	0.27	0.07
Ethnic group											
**Hoa**	23	0.39	0	0.17	0.13	0.22	0.61	0.00	0.13	0.39	0.09
**Kho Me**	1	—	—	—	—	—	—	—	—	—	—
**Kinh**	399	0.41	0.02	0.04	0.23	0.35	0.59	0.01	0.23	0.27	0.08
**Mong**	115	0.29	0.13	0.07	0.04	0.09	0.71	0.05	0.41	0.16	0.09
**Nung**	21	0.56	0.05	0.14	0.3	0.37	0.44	0.05	0.10	0.24	0.05
**Tay**	62	0.45	0.07	0.05	0.3	0.33	0.55	0.02	0.16	0.30	0.07
**Thai**	1	—	—	—	—	—	—	—	—	—	—

However, remarkable geographic differences in haplogroup frequencies can be observed (Fig. 1B). For instance, haplogroup composition in the northern locations of Cao Bang, Ha Noi, and Hai Phong is quite similar, with haplogroup M being the most frequent (≥42% in all these locations) and M7 its main sub-haplogroup (≥20%), followed by haplogroup R9’F with frequencies between 28-36% (Table 2). The exception to this pattern in the North is Lao Cai, where there is a high frequency of haplogroup N (71%), with haplogroup B (41%) being its main sub-clade followed by R9’F (16%); moreover, the frequency of haplogroup C is the highest in the whole data set (13%). As mentioned above, the distinctive character of Lao Cai derives from the fact that our sample from this region is made up primarily by the Mong ethnic group, which shows a pattern of mtDNA variation that cannot be taken as fully representative of the whole population of this region. As with the Mong, the Hoa mtDNAs have also some peculiarity with respect to the rest of ethnic groups studied. For instance, the frequency of R9’F (39%) is substantially higher than the average of the rest of the sample (27% on average for the whole of Vietnam).

In order to facilitate interpretation of haplogroup frequencies between different locations, haplogroups were collapsed into categories that represent the main SEA phylogenetic branches (namely, A, B, C, D, R9’F, M, M7, and N), and these frequencies were interpolated into geographic maps (Fig. 2B and C). The maps show that M and M7 show the highest frequency in the Northeast of the country following the coast along the Gulf of Tonkin and the lowest frequencies in the northern West and the southern extreme of Vietnam (Mekong delta), where haplogroup N is more common. Haplogroups A and C are more frequent in the northern West of Vietnam (Lao Cai), with the lowest values in the South. Haplogroups B reaches the highest frequency in the Northwest, and the lowest values are found also in the Northeast (Red River delta). It is in the South, in Ho Chi Minh, that haplogroup D reaches the highest frequency (9%). Finally, the interpolated map of haplogroup R9’F shows also a fragmented frequency distribution pattern: it reaches its highest frequency in the Red River delta (32–36%), the frequency decreases towards the Northwest (16% in the neighboring region of Lao Cai) and in the center of the country (21% in Da Nang), but rises again in the Mekong River delta (29% in Ho Chi Minh).

Networks of CR sequences were built (Figure S1) to investigate possible clusters of haplotypes attributed to particular ethnic groups. The trees show a number of haplotypes that are shared between ethnicities along different branches of the phylogeny, compatible with historical gene flow between them.

An interesting haplotype feature deserves particular mention. Wen and collaborators^48^ found in Chinese samples the CR motif T16189C–T16217C–C16261T–T16357C, which was claimed to be exclusive of the Hmong-Mien speaking populations who live in the South of China. Our results support this hypothesis, as we have also found this motif in six samples of Vietnamese Mong people, which are known as Hmong/Miao in China.

### Analysis of mitogenomes and identification of new phylogenetic lineages

Among the CR Vietnam sequences analyzed in the present study, we identified two distinctive motifs that had hitherto remained uncharacterized. The current version of the reference mtDNA haplogroup nomenclature (PhyloTree Build 17) does not contain these phylogenetic branches, and therefore new names were given to them. Confirmatory information of these sequence motifs could be obtained from the analysis of the mitogenomes obtained from 1000G and the literature, especially from^49^. Mitogenomes added phylogenetic support and resolution to these new clades.

**Figure 3:**
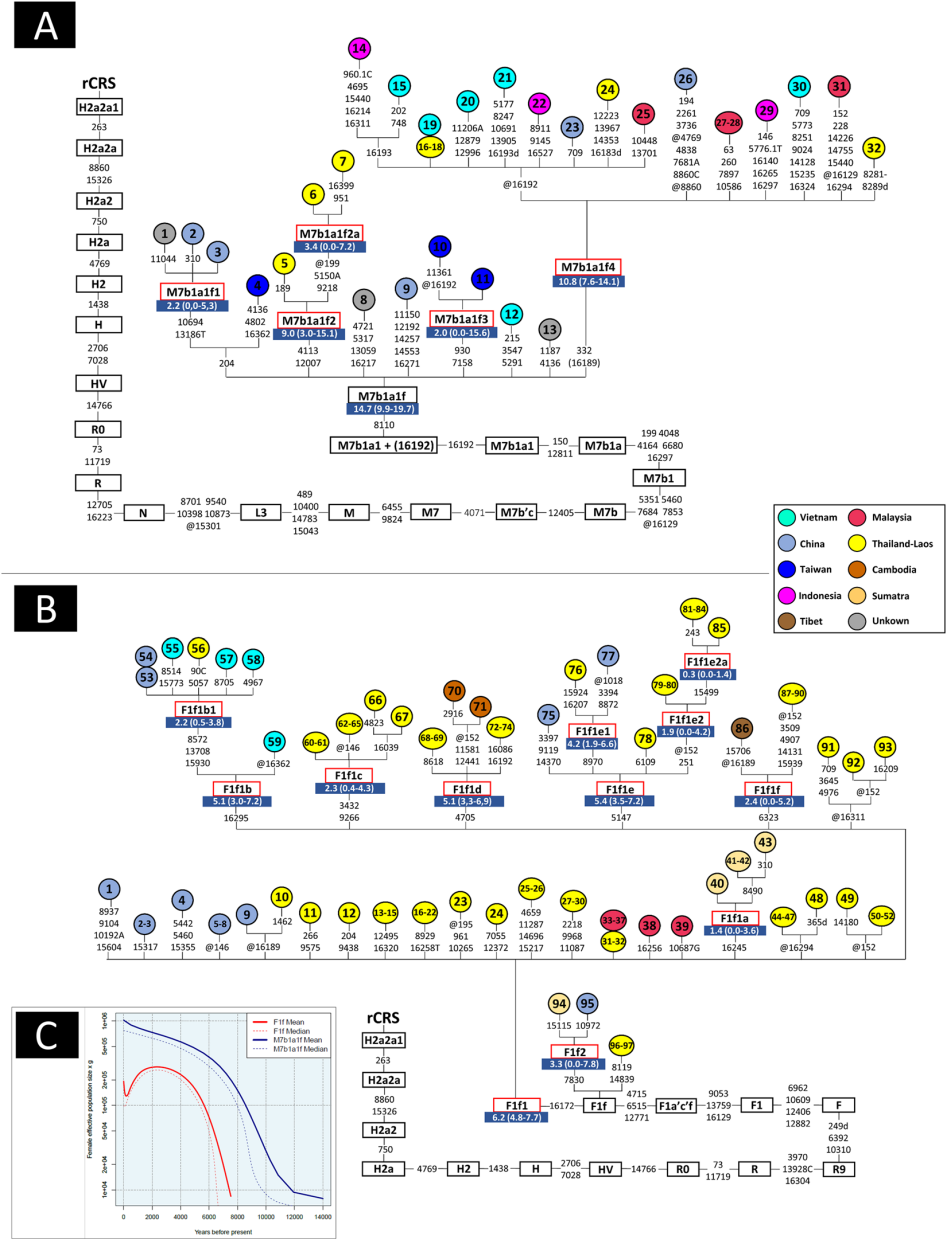
Maximum parsimony trees based on mitogenomes representing haplogroup M7b1a1f (**A**) and F1f (**B**). The revised Cambridge reference sequence (rCRS) is shown as reference for nomenclature^52^. Genetic variants are indicated along the branches of phylogeny as follows: all of them are transitions unless a suffix A, C, G, or T indicates a transversion, and a prefix ‘@’ indicates a back mutation. As per common practice, the trees do not consider hotspot mutations at positions 16182, 16183, and 16519, nor variation around position 310 and length or point heteroplasmies. The ID numbers in the tips of the phylogeny identify mitogenomes as indicated in Table S2; this table also show details of the geographic or ethnic origin of all the samples. **(C)** EBSPs of haplogroup F1f and M7b1a1f obtained from complete mitogenomes. EBSPs with 95% HPD (highest posterior density) intervals are provided in Figure S3.

We first focused our attention on the CR motif observed in a number of haplotypes characterized by transition C332T (1.7% of the haplotypes in the major ethnicity, the Kinh). Transition C332T is very uncommon, e.g. it does not appear in the phylogeny from Phylotree (see Weissensteiner *et al*.^24^) and it has no mutational hits in Soares *et al*.^29^. A total of five mitogenomes carrying C332T were found in the 1000G project Vietnamese samples (#HG02031, #HG02121, #HG02079, #HG01840, and #KU131379) (Fig. 3A). The five mitogenomes share also mutation T8110C plus all the mutations that led from the rCRS to haplogroup M7b1a1 + (16192) according to Phylotree Build 17. An additional mitogenome from Vietnam (#HG02141) carries all the characteristic mutations of M7b1a1 + (16192) with the exception of variant C332T (Fig. 3A). The mitogenomes sharing C332T share also variant T16189C. Taking into account the information available from the complete genomes, we added a new clade to the phylogeny defined by the motif C332T–(T16189C) and named it M7b1a1f4. The parenthesis indicates that variant T16189C is not always present in members of this clade, as testified from the CR data carrying C332T (this site has a high mutation rate^24,29^). By inspecting other mitogenomes from the literature, we detected 14 additional M7b1a1f4 haplotypes sampled in the Indochinese Peninsula, including Cambodia, Malaysia and Indonesia. In addition, there are 13 other mitogenomes belonging to M7b1a1f that do not carry C332T but other extra motifs, thus constituting other minor sub-clades. Again, these other genomes were sampled in different locations of the Indochinese Peninsula. The analysis of control region sequences belonging to haplogroup M7b1a1f reveals roughly the same pattern observed for the mitogenomes, although indicating a higher prevalence of Vietnamese sequences within sub-clade M7b1a1f4. By revising the literature on CR sequences, we could detect only a few M7b1a1f4 members in populations from SEA ancestry and always at very low frequencies, most of them in Malay^50,51^. By examining control region sequences belonging to M7b1a1f, we observed that variant T16189C is not always present in M7b1a1f4 haplotypes carrying C332T. Moreover, a few M7b1a1f4 haplotypes, only present in Vietnam, carry T16324C (Fig. 3A). From a large worldwide database of control region sequences, it could be corroborated that M7b1a1f and its sub-clades are almost restricted to the Indochina Peninsula (Figure S2A). In Vietnam, these haplotypes were found to be carried almost exclusively by people belonging to the major ethnic group (Kinh). M7b1a1f is an old clade (TMRCA: 14.7 kya [9.9–19.7]), as is its sub-clade M7b1a1f4 (TMRCA: 10.8 kya [7.6–14.1]).

Another singular sequence motif observed in our samples from Vietnam falls within haplogroup F1f (Fig. 3B). Thus, the double mutations T16172C–C16295T plus all the characteristic mutations that led to F1f are shared by approximately 3% of the haplotypes within the Kinh. The search for individuals belonging to F1f reveals a more complex phylogeny than expected. There are 97 complete genomes gathered from the literature and databases; most of them carry variant T16172C, then forming a sub-clade named here as F1f1. Within F1f1 there are six different sub-clades, all of them characterized by one or two quite stable transitions. One of these clades is defined by C16295T, named as F1f1b, and four out of seven mitogenomes were sampled in Vietnam, with the other three in neighboring countries. This sub-clade is, in fact, the only one within F1f represented by samples from Vietnam. The CR data available from Vietnam fall entirely within F1fb, revealing also that this clade has very little variation (Figure S2B). The rest of the data belonging to F1f is well represented in Thailand and Laos. TMRCA for F1f is only 6.2 kya (4.8–7.7); while for F1f1b it is 5.1 kya (3.0–7.2).

### AMOVA analysis of mtDNA profiles

AMOVA was conducted by classifying the sample by sampling locations, ethnic groups and broad geographic regions. As expected, within genetic variation accounted for most of the variation, ranging from 98.08–99.69% (Table 3), independently of the sub-division considered. Within population variation was higher when considering sample location (98.08%), and this pattern was confirmed when the data generated in the present study was meta-analyzed with the data compiled from the literature (98.37%).

**Table 3:** Analysis of molecular variance (AMOVA) accounting for main geographic regions (MGR), ethnic group (EG), and sampling location (SL) (*P*-value < 0.0000).

Source of Variation	Percentage of variation					
	Present study			Meta-analysis		
	MGR	EG	SL	MGR	EG	SL
Among groups	0	0.57	1.61	0	1.28	1.31
Among population within groups	0.31	0.03	0.31	0.41	0.2	0.31
Within populations	99.69	99.41	98.08	99.59	98.52	98.37

### Principal Component Analysis

**Figure 4:**
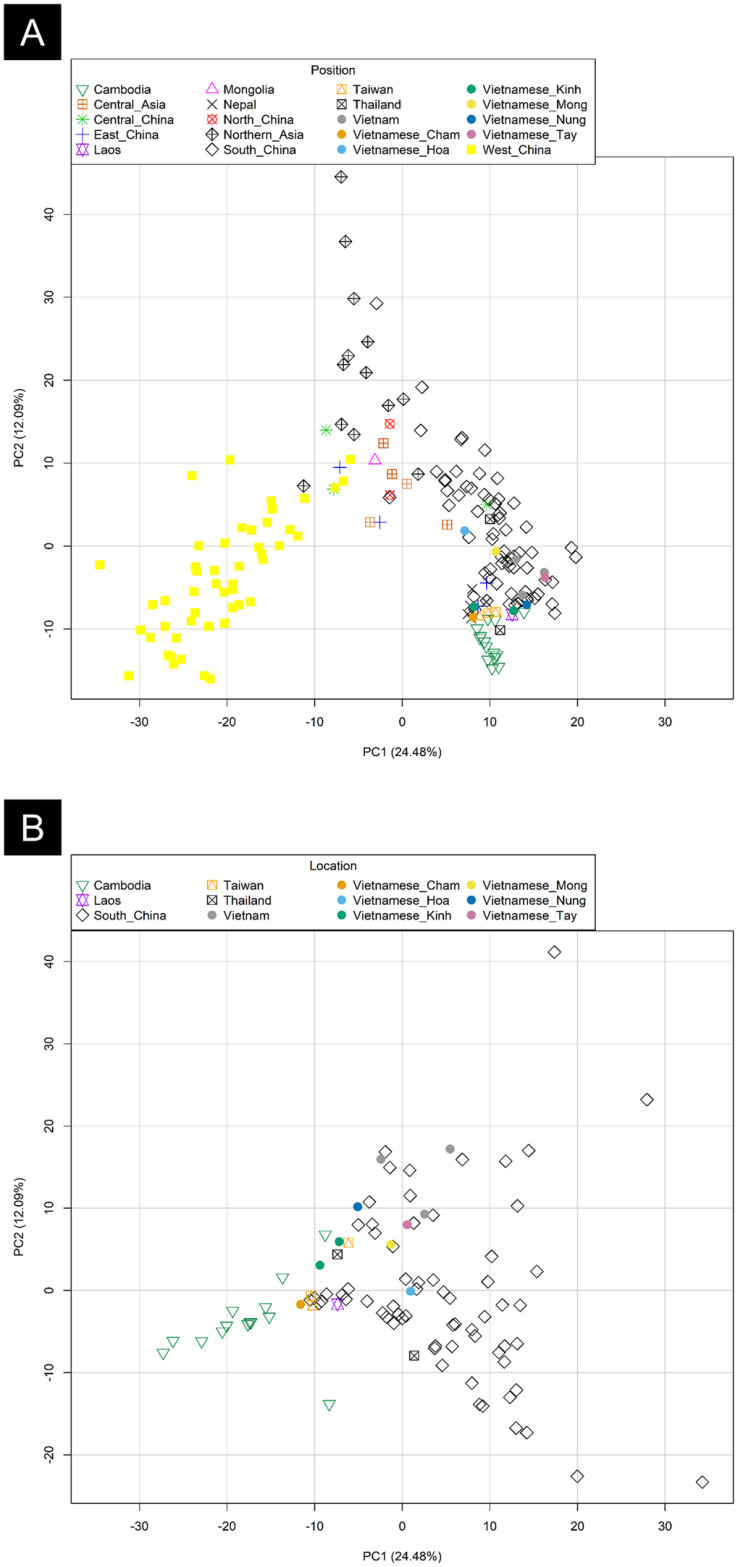
PCA of Vietnamese populations analyzed in the present article *versus* other Asian populations **(A)** and *versus* SEA/Southern China populations **(B)**. Haplogroup frequencies from the reference populations were taken from Zhang *et al*.^16^. Note that there are two Vietnam_Kinh samples in the plot, one represents our sample from Kinh and another one that was taken from the literature.

PCA based on haplogroup frequencies allows visualizing global patterns of variation between populations. We conducted a PCA based on mtDNA haplogroup frequencies of 180 Asian populations (Fig. 4A).

The PCA displays an outstanding clustering of population samples by main geographic areas. The first component (PC1), which explains ~24% of the genetic variance, separates all the population samples in three main areas of the plot, with the North China population samples in one pole, and the opposite pole made up by population samples from the Indochinese Peninsula (including all the ethnic groups from Vietnam, Laos, Thailand, etc) and Eastern Asia (including a number of western Chinese samples plus Taiwan). A more geographically heterogeneous group of different populations from northern and Central Asia fall in between. Turning to the second component (PC2), which explains ~12% of the variance, the most remarkable feature is that it splits the SEA populations from those in western Asia (Fig. 4A). The patterns observed in this PCA are in good agreement with previous results (e.g. Tabbada *et al*.^52^).

In order to further investigate the patterns of haplogroup variations in Vietnamese populations we performed a second PCA (Fig. 4B), this time restricting the analysis to South China and SEA populations. The ethnic groups Thai and Kho Me were omitted from these analyses due to their small sample size. In the PC1, Vietnamese populations appear scattered together with samples from South China and Thailand. Curiously, the Cambodia samples cluster in the opposite end of the plot. The PC2 however indicates a more pronounced genetic proximity between the Cambodian samples and those from Vietnam. A few populations from South China appear as clearly differentiated in the plot.

### Extended Bayesian Skyline Plots of haplogroups and populations

F1f1 shows a population growth beginning at ~8 kya, followed by a slight decrease starting at ~2 kya but dramatically descending from ~1 kya onwards. M7b1a1f however, appears ~14 kya and it experiences a continuous growth until the present. The coalescent times obtained by EBSPs for lineages F1f1 and M7b1a1f coincide with TMRCA obtained using ML (Fig. 3C; Figure S3).

EBSPs were also inferred from ethnic groups (Figure S4). The most outstanding feature is the demographic pattern of the Cham, which is rather similar to that of haplogroup F1f1. Tay and Kinh show a moderate historical growth until the present day, while other groups show a more constant *N*_*e*_ through time (although the Hoa and the Nung with large credibility regions).

### Maternal gene flow

Migration rates were firstly analyzed by linguistic families. The full migration model was the most probable (Table 4); it shows very different population sizes as well as asymmetric gene flow between the main linguistic groups in Vietnam (Table S4). The number of immigrants per generation (*N*_*m*_) was estimated from the data. The Tai-Kadai family showed the highest migration rates towards the Austroasiatic family (*N*_*m*_ = 214; mainly represented by the King ethnic group). The Hmong-Miao family showed substantial levels of migration towards the Tai-Kadai (*N*_*m*_ = 164). Finally, lower rates of gene flow were obtained from Austroasiatic and Tai-Kadai to Cham family (*N*_*m*_ = 10 and *N*_*m*_ = 25, respectively). No maternal gene flow was detected between the other linguistic pairs analyzed (Table S4).

**Table 4:** Maternal gene flow between the main linguistic families within Vietnam, and between Vietnam neighboring countries.

Model	lmL	LBF	Probability
Linguistic groups			
Full	−4003.198	−168.8531	0.000
Panmictic	−4087.624	0.000000	1.000
Vietnam vs Laos			
Full	−2725.178	−16.93701	0.000
Panmictic	−2716.710	0.00000	1.000
Laos to Vietnam	−2726.531	−19.64204	0.000
Vietnam to Laos	−2763.987	−94.55403	0.000
Vietnam vs Cambodia			
Full	−2332.827	−5.512706	0.004
Panmictic	−2358.694	−57.246388	0.000
Cambodia to Vietnam	−2333.580	−7.018030	0.001
Vietnam to Cambodia	−2330.071	0.000000	0.995

Secondly, gene flow was examined between Vietnamese and neighboring populations in Cambodia and Laos. The most probable gene flow model for the pair Cambodia-Vietnam was a unidirectional migration pattern from Vietnam to Cambodia (Table 4; Table S4; *N*_*m*_ = 22).

For the population pair Laos-Vietnam, however, the panmictic gene flow model was the most probable (Table 4). This finding suggests that these two populations represent effectively a single population unit, both being part of the same panmictic unit and therefore pointing to high levels of gene flow between them.

### Genome-wide analysis of Vietnamese

**Figure 5:**
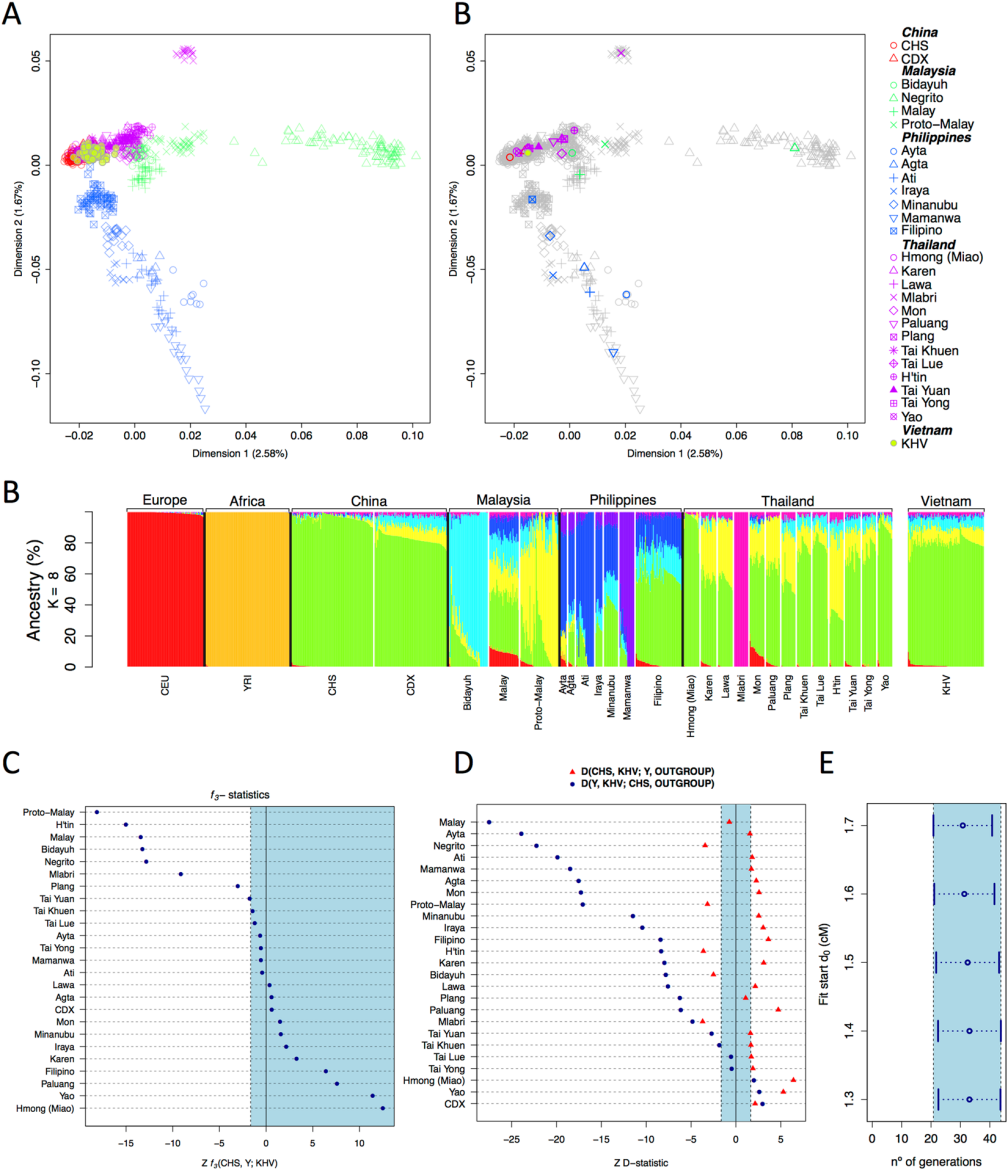
Analysis carried out on autosomal SNPs. **(A)** MDS of population samples from the Indochinese Peninsula and neighboring samples. Both plots were built using the same sample sets, but the one to the right aims at highlighting the center of each population sample points in order to easy interpretation **(B)** Admixture analysis including reference samples from Europe (CEU) and Africa (YRI). **(C)** Analysis of *f3*-statistics of Vietnamese (KHV) *versus* different neighboring population samples. **(D)** *D*-statistics of Vietnamese built as follows *D*(CHS, KHV; Y, OUTGROUP) and *D*(Y, KHV; CHS OUTGROUP). **(E)** Estimates of admixture between Chinese and Malay, using the samples CHS and Malay as subrogates of those that contributed to the present genomic architecture of present-day Vietnamese. Estimates were statistically significant according to the *ad hoc* z test from ALDER.

An initial MDS analysis computed on IBS values indicates that all Asian populations fall in the same cluster, clearly separated from the European and the African poles (Figure S5). A second MDS analysis was carried out using only populations from SEA in order to disentangle clusters of genetic variation within this region (Fig. 5A). Dimension 1 highlights the distinctive features of the Negrito from Malaysia, although the other populations from this region cluster very close to the Vietnamese (KHV). Dimension 2 separates Philippines from the China-Thailand-Malaysia group (including Negrito). The Mlabri from Thailand appear also differentiated from the main group in this dimension.

Admixture analysis indicates that, for the best cross validation value (*K* = 8), the KHV population has a main component that is most prevalent in the Chinese, and two minor components that find the highest frequencies in the Bidayuh from Malaysia and the Proto-Malay, suggesting a close genetic proximity between Chinese and Malaysians (Fig. 5B).

The *f3*-statistic, built as *f3*(CHS,Y;KHV), indicates that the sample KHV can be explained by a mixture of Chinese and other populations from SEA, especially the Proto-Malay and other populations from Malaysia (Bidayu, Malay, and Negrito) and/or from Thailand (H’tin and Mlabri) (Fig. 5C). The *D*-statistic, built as *D*(Y, KHV; CHS, OUTGROUP), indicates that the Chinese (represented by CHS) have an unquestionable contribution to KHV, and this is statistically significant using almost all SEA populations as reference (Fig. 5D). However, *D*(Y, KHV; CHS, OUTGROUP) suggest that only a few populations from SEA seem to statistically contribute to KHV; again the Malaysians (represented by the Proto-Malay, the Negrito, and the Bidayut) together with the Thai (represented by the Mlabri and the H’tin) are the samples that could be admixed with Chinese in the Vietnamese gene pool. The fact that the negative values are much higher in *D*(Y, KHV; CHS, OUTGROUP) than in *D*(Y, KHV; CHS, OUTGROUP) reveals that the Chinese component has a more clear presence in KHV. This result is in good agreement with ADMIXTURE.

ALDER was used to estimate the time of admixture, which was shown to be statistically significant under an *ad hoc* z test (Fig. 5E). The best estimates point to an admixture event occurring about 32 (21–44) generations ago or ~800 ya (assuming 25 years per generation).

## Discussion

Overall, the genetic variation observed in the Vietnamese fits well with mtDNA patterns observed in SEA, which is considered the most diverse and polymorphic region of the continent^6^. As expected, mtDNA diversity is very high across the Vietnamese territory.

The majority of Vietnamese people carried mtDNA haplotypes that clustered in clades M7 (20%) and R9’F (27%), two major maternal lineages dominating not only Vietnam, but SEA more generally. Other sub-haplogroups, such as A, B, C and D are represented in the Vietnam territory but with lower frequencies, with the exception of haplogroup B in Lao Cai, where it reaches 41% of the total. Haplogroup M is more prevalent in the North and in the Center of the territory than in the South. The high frequency of its sub-branch M7 is in good agreement with a previous study from 2002, which found this haplogroup only in the southern part of the East Asia, in countries such as Korea or Japan^53^. M7 is very rare in Central Asia, and the estimation of coalescence time observed by Kivisild *et al*.^53^, could reflect a re-population of the Southern Area of Asia that occurred after the Last Glacial Maximum. R9’F reaches high frequencies all across Vietnam, with the lowest values observed in Lao Cai (16%). Haplogroup frequencies observed in previous studies fit well with the values observed in the present study. The analysis by Irwin *et al*.^14^ in Ha Noi City population indicated a high R9’F representation (>27%) followed by B and M7 (20%), and low frequency of haplogroups, N, A, C and D (<5%).

The fact that there are over 50 recognized ethnic groups distributed in the highlands has also contributed to the high genetic variability observed. The geographic and cultural characteristics of Vietnam configure a scenario that does not favor random mixing between ethnic groups; furthermore, some of these groups divide their population into social echelons that could restrict genetic exchange (UK Embassy of Vietnam http://www.vietnamembassy.org.uk/population.html; accessed June 2017). Our data were sampled from different ethnic groups, and therefore it is possible to make inferences about genetic exchange between them and explore the population sub-structure of the country. AMOVA of Vietnamese groups detects only moderate population stratification, and the variation is lower within ethnic groups than within samples sorted by geographic region. Since inferences based on Wright’s *F*_*ST*_ alone can be misguided^54,55^, evaluation of stratification has to be examined from different angles. Thus, phylogenetic, phylogeographic, and MDS analyses of Vietnamese populations reveal the existence of more notable stratification in the country; at the same time, these analyses also provide evidence for historical gene flow between ethnic groups. Within this scenario, the most distinctive group is the Mong population (Lao Cai). Their mtDNA composition is quite different from those of neighboring ethnic groups from North Vietnam (Kinh, Nung, and Tay). The results find additional support in historical and anthropological data: archaeological evidence indicates that the early Mong were linked with the Neolithic cultures that settled in the Middle Reach of the Yangtze River and in Central-southern China, while linguistic evidence suggests that Mong occupied the same areas of southern China for at least the past 2,000 years^56^. Wen *et al*.^48^ indicated that most of Mong mtDNA lineages have their origin in southern China, although Mong populations seemed to have had contact with northeast Asians (Chinese Han).

Specific analysis of migration patterns also supports the conclusion that ethnic groups have been permeable to gene flow, and therefore we should assume that the effect of geographic barriers in gene flow exchange between regions has been moderate. Population groups belonging to the Tai-Kadai linguistic family show the highest values of migration rates towards the Austroasiatic family groups, probably reflecting an assimilation of Tai-Kadai mtDNA lineages by the Austroasiatic family. This seems to agree well with the famous Austro-Tai hypothesis initially put forward by the anthropologist Paul King Benedict^57^, which proposes that the Tai-Kadai and Austronesian languages from southern China and the Pacific are closely related.

Historical demographic events were also examined by way of EBSPs. The plot obtained from haplogroup F1f shows the existence of important demographic changes occurring about 1,000 years ago. Also, analyses carried out in ethnic groups indicate a similar demographic hallmark in the Cham ethnic group. Interestingly, the observed abrupt change of *N*_e_ detected in the mtDNA sequences coincides in time with one of the most important historical demographic events occurring in Vietnam, the so called “southern expansion” (known as Nam tiến) from their original heartland in the Red River Delta (North of present day Vietnam), following the coast. Historical documents indicate that this expansion began in the 10–11^th^ centuries and lasted about 700 years until the mid-18^th^ century. The colonization process initiated by Vietnam was justified by geographic and demographic reasons. This expansion involved intense wars with the Chams and left the population of Vietnam dramatically reduced. It was during the 17–19^th^ centuries that the Vietnamese penetrated the Mekong Delta in the South. Not all the ethnic groups analyzed in the present study show the same demographic pattern in EBSPs, which suggests that most likely the Nam tiến did not have the same impact in all Vietnamese. Estimates of migration rates indicate that the Cham neither were assimilated nor participated in gene exchange with other groups, overall indicating that the dramatic reduction of Champ *Ne* did not involve assimilation by other populations; see also^17^. Migration rates also indicate the existence of important assimilation of the Hmong-Miao people by the Tai-Kadai family group, both family groups coinciding geographically in the North of the country.

We have examined phylogeographic and gene flow patterns with other neighboring countries. PCA based on mtDNA haplotypes suggests a close genetic proximity between different Vietnamese subpopulations and other Southern China and SEA populations. Phylogeographic characteristics of M7b1a1f and F1f1 suggest that genetic exchange has been common in ancient but also more recent times.^1,48,58^. Migration rates estimated from mtDNA data also agree well with historical records. The data show a unidirectional gene flow from Vietnam to Cambodia, a finding that fits with the historical incursions of Vietnamese into Cambodian territories already in the Dark Ages of Cambodia (from the 16^th^ to 19^th^ centuries), soon after the collapse of the Khmer Empire. These movements began with penetration of the Vietnamese Kinh into the southern Mekong Delta, displacing the Khmers^59^, and giving way to a period in which Cambodia was alternately controlled by Thailand (previously named Siam) and Vietnam. Nowadays Vietnamese people represent the second largest minority in Cambodia, concentrated mostly in the country.

The high genetic similarity between Laotians and Vietnamese was previously noted by Bodner *et al*.^12^. These authors did not find significant differences in mtDNA patterns between these populations, suggesting intense gene flow by migration between them. These movements may have been favored by their geographic proximity as well as by the partly shared political history of both countries. Our analysis shows a panmitic migration model is the most likely, hence adding further support to this hypothesis. In addition, the Vietnamese language (Kinh ethnicity) belongs to a branch of the Austroasiatic language family with an unexpected similarity with that of the Laotian population, despite the fact that Laos is mainly dominated by Daic language family (Tai-Kadai family)^12^. This might indicate that a great proportion of Austroasiatic maternal lineages were assimilated by the Laos population.

With the aim of contrasting phylogenetic and demographic patterns observed with mtDNA data, we also investigated genome-wide SNP patterns using data from Vietnam and neighboring countries. MDS analysis indicates a close proximity of the Vietnamese Kinh (KHV) with Chinese, Thai and Malaysians. This relation is also evident when examining admixture patterns, which indicate that the presence of the Chinese component is more prevalent in Vietnamese. *F3*-statistics and *D*-statistics give statistical support to this two-way admixture. A rough estimate based on genome-wide data fits surprisingly well with the results obtained from mtDNA data and a different set of samples from Vietnam, by dating the time of admixture to the Nam tiến expansion.

The results of the present study confirm the existence of high molecular variation in Vietnam, as expected from a region considered as one of the main cradles for Asian settlements. Notwithstanding the moderate genetic differences existing between regions and ethnic groups in Vietnam, there is evidence for important gene flow within the country and more broadly within the Indochinese Peninsula. The data presented in the present study is the largest dataset generated to date; it is of interest not only in anthropological studies, but also in other applied fields of medical research; e.g. forensic genetics, where databases of mtDNA data are needed to estimate the weight of the evidence in casework and kinship analysis^60^. Haplogroup frequencies estimated from this sampling strategy are also key for the interpretation of biomedical studies, such as case-control studies where undetected population structure could lead to undesirable false positive of association with diseases^61,62^. From an anthropological point of view, the overall scenario is that present-day Vietnamese have a dual ethnic origin: a major component coming from South of China, superimposed to a minor component originating from a Thai-Indonesian composite. The Nam tiến has probably been key for the configuration of the genome architecture of present-day Vietnamese.

## Electronic supplementary material

Table S1

Table S2

Table S3

Table S4

Supplementary Data
