# Supplementary material for: Phylogeographic and genome-wide investigations of Vietnam ethnic groups reveal signatures of complex historical demographic movements
Source: Sci Rep. 2017 Oct 3;7:12630. doi: 10.1038/s41598-017-12813-6 (PMC5626762; doi:10.1038/s41598-017-12813-6)
Supplement: Supplementary file 5 — Supplementary Data [file 41598_2017_12813_MOESM5_ESM.pdf]

Phylogeographic and genome-wide investigations of Vietnam ethnic groups reveal signatures of complex historical demographic movements

Pischedda S, Barral-Arca R, Gómez-Carballa A, Pardo-Seco J, Catelli ML, Álvarez-Iglesias V, Cárdenas JM, Nguyen ND, Ha HH, Le AT, Martinón-Torres F, Vullo C, Salas A

Supplementary Data

Figure S1. Phylogenetic networks of HVS-I mtDNA Vietnamese sequences.
Figure S2. Maximum parsimony trees based on CR sequences representing haplogroup M7b1a1f (A) and F1f (B). See Figure 3 for more information.
Figure S3. EBSPs (with 95% CI) of haplogroup F1f and M7b1a1f obtained from complete mitogenomes.
Figure S4. EBSP of main Vietnamese ethnics.
Figure S5. MDS plot of IBS values considering different SEA populations (Table S2) and one European (CEU) and one African population (YRI).

Figure S1.

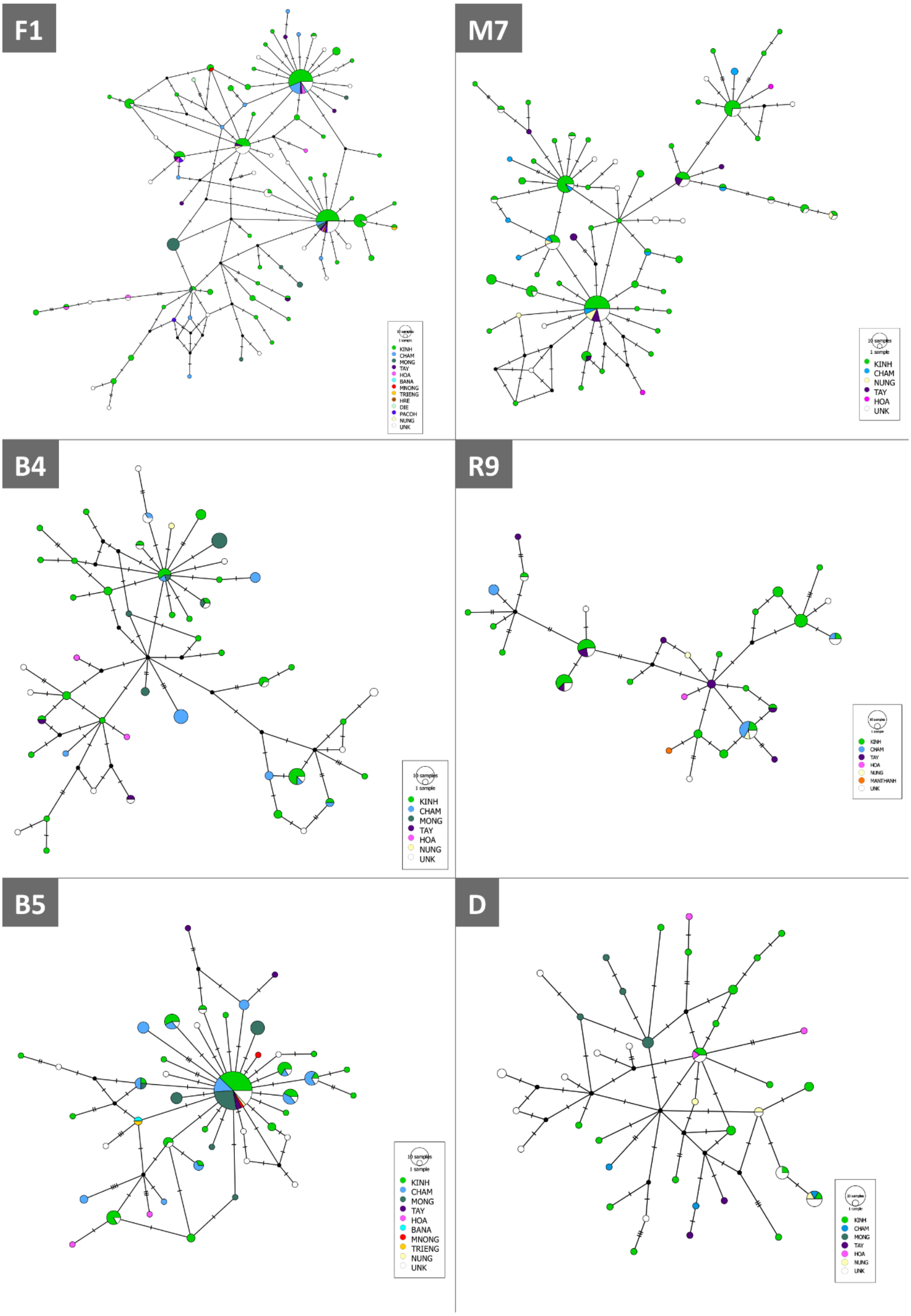
F1
KINH
CHAM
MONG
TAY
HOA
BANA
MNONG
TRIENG
HRE
DIE
PACOH
NUNG
UNK
M7
KINH
CHAM
NUNG
TAY
HOA
UNK
B4
KINH
CHAM
MONG
TAY
HOA
NUNG
UNK
R9
KINH
CHAM
TAY
HOA
NUNG
MANTHANH
UNK
B5
KINH
CHAM
MONG
TAY
HOA
BANA
MNONG
TRIENG
NUNG
UNK
D
KINH
CHAM
MONG
TAY
HOA
NUNG
UNK

Figure S1.

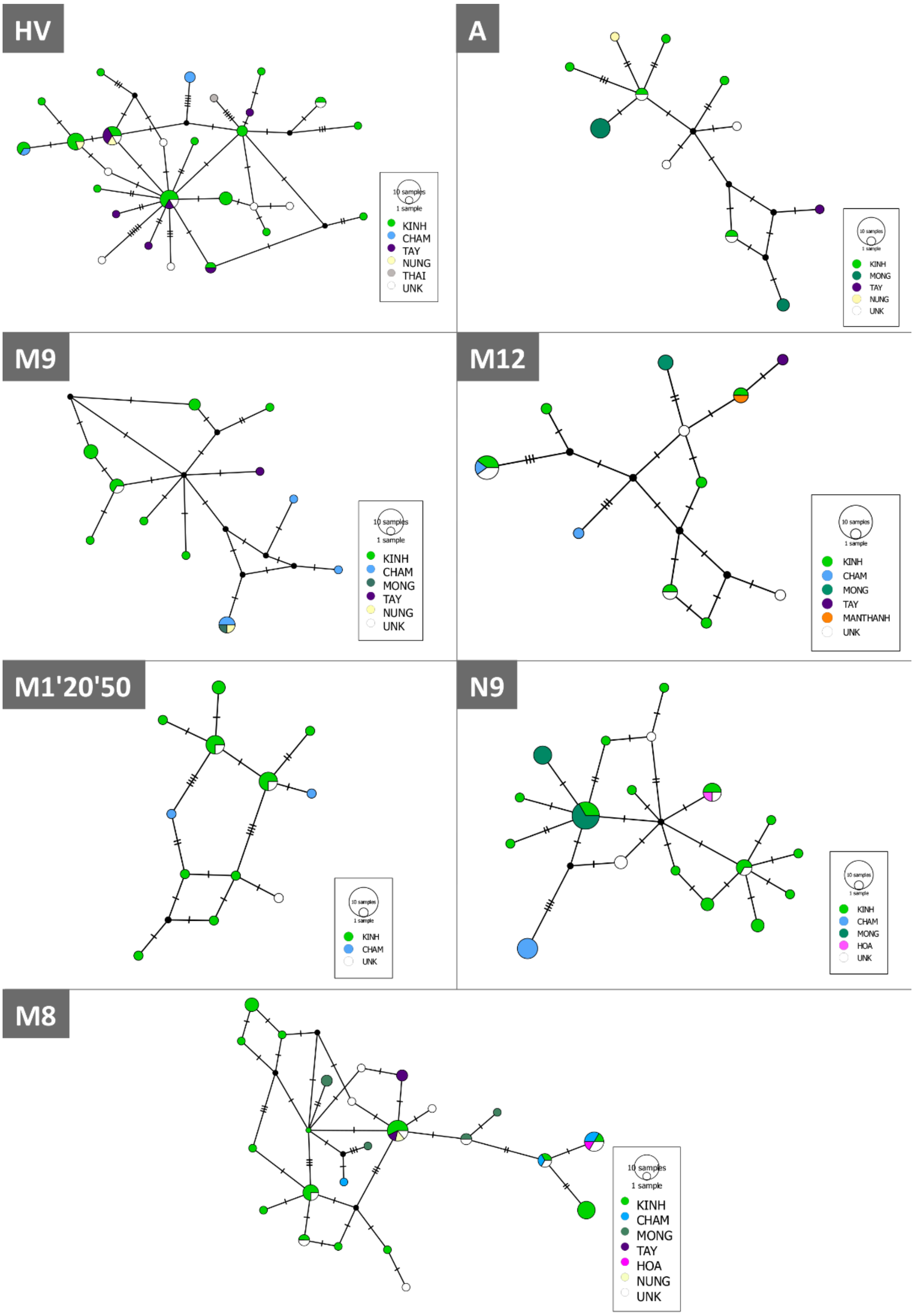
HV
10 samples
1 sample
KINH
CHAM
TAY
NUNG
THAI
UNK
A
10 samples
1 sample
KINH
MONG
TAY
NUNG
UNK
M9
10 samples
1 sample
KINH
CHAM
MONG
TAY
NUNG
UNK
M12
10 samples
1 sample
KINH
CHAM
MONG
TAY
MANTHANH
UNK
M1'20'50
10 samples
1 sample
KINH
CHAM
UNK
N9
10 samples
1 sample
KINH
CHAM
MONG
HOA
UNK
M8
10 samples
1 sample
KINH
CHAM
MONG
TAY
HOA
NUNG
UNK

Figure S2.

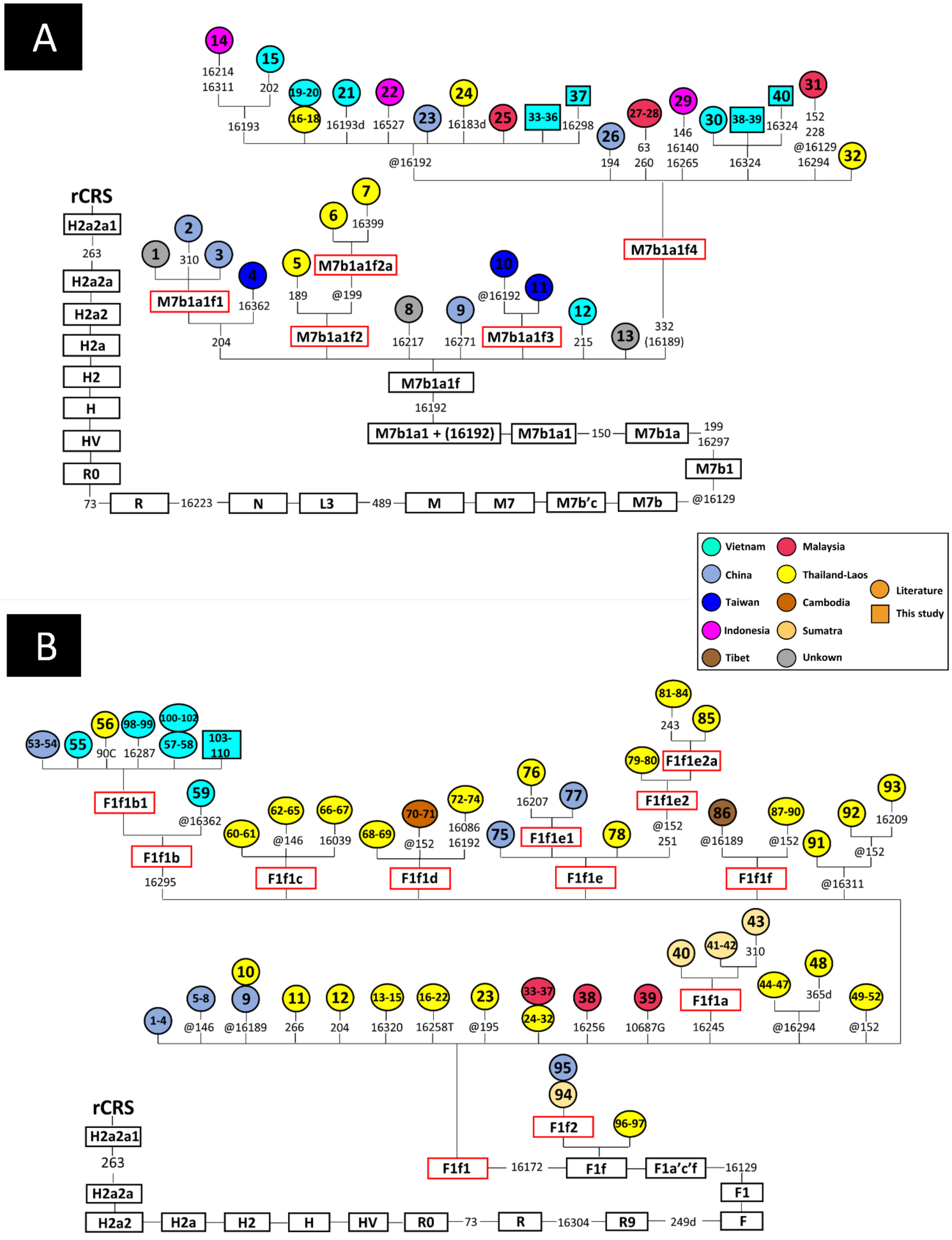
A
rCRS
H2a2a1
263
H2a2a
H2a2
H2a
H2
H
HV
R0
73
R
16223
N
L3
489
M
M7
M7b'c
M7b
@16129
M7b1
199
16297
M7b1a
150
M7b1a1
M7b1a1 + (16192)
16192
M7b1a1f
M7b1a1f1
M7b1a1f2
M7b1a1f2a
M7b1a1f3
M7b1a1f4
204
310
16362
189
@199
16399
16217
16271
@16192
215
332
(16189)
16214
16311
202
16193
16193d
16527
16183d
16298
63
260
194
146
16140
16265
16324
152
228
@16129
16294
B
rCRS
H2a2a1
263
H2a2a
H2a2
H2a
H2
H
HV
R0
73
R
16304
R9
249d
F
F1
16129
F1a'c'f
F1f
16172
F1f1
F1f2
F1f1a
F1f1b
F1f1b1
F1f1c
F1f1d
F1f1e
F1f1e1
F1f1e2
F1f1e2a
F1f1f
16295
@16362
90C
16287
@146
16039
@152
16086
16192
16207
243
251
@16189
16209
@16311
@16189
266
204
16320
16258T
@195
16256
10687G
16245
310
365d
@16294
Vietnam
China
Taiwan
Indonesia
Tibet
Malaysia
Thailand-Laos
Cambodia
Sumatra
Unkown
Literature
This study

Figure S3.

A

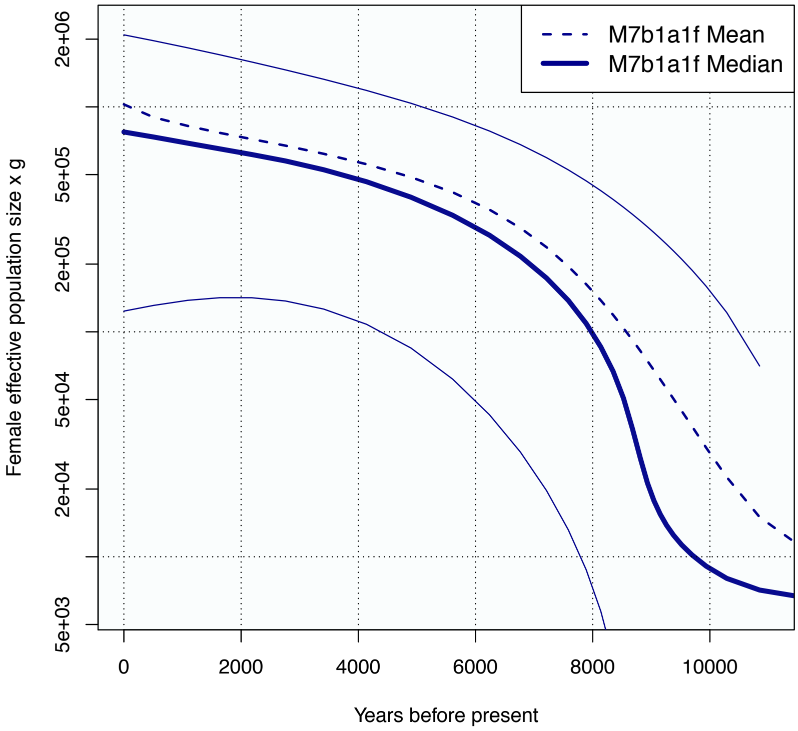

M7b1a1f Mean
M7b1a1f Median
Female effective population size x g
2e+06
5e+05
2e+05
5e+04
2e+04
5e+03
0
2000
4000
6000
8000
10000
Years before present

B

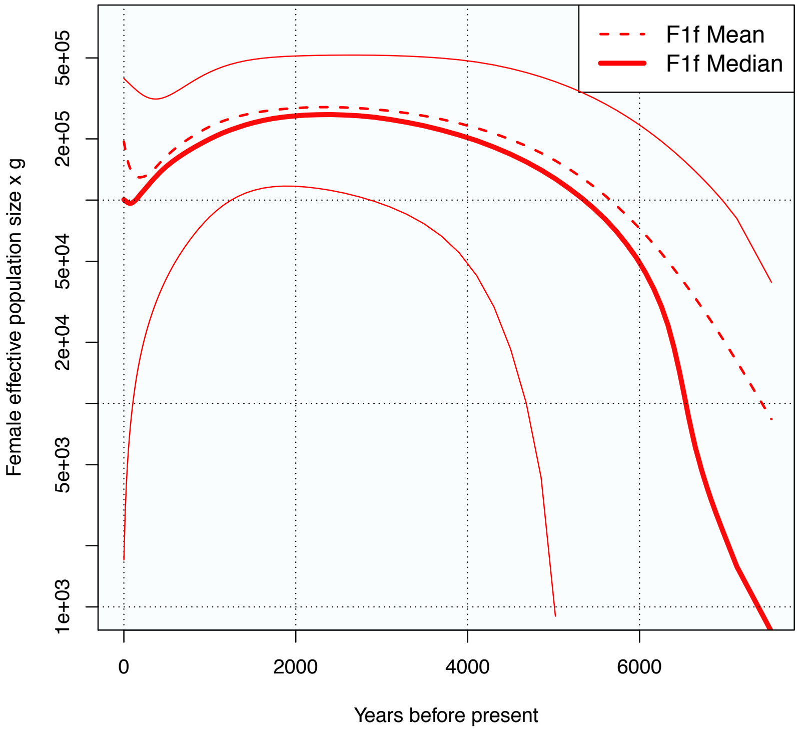

F1f Mean
F1f Median
Female effective population size x g
5e+05
2e+05
5e+04
2e+04
5e+03
1e+03
0
2000
4000
6000
Years before present

Figure S4.

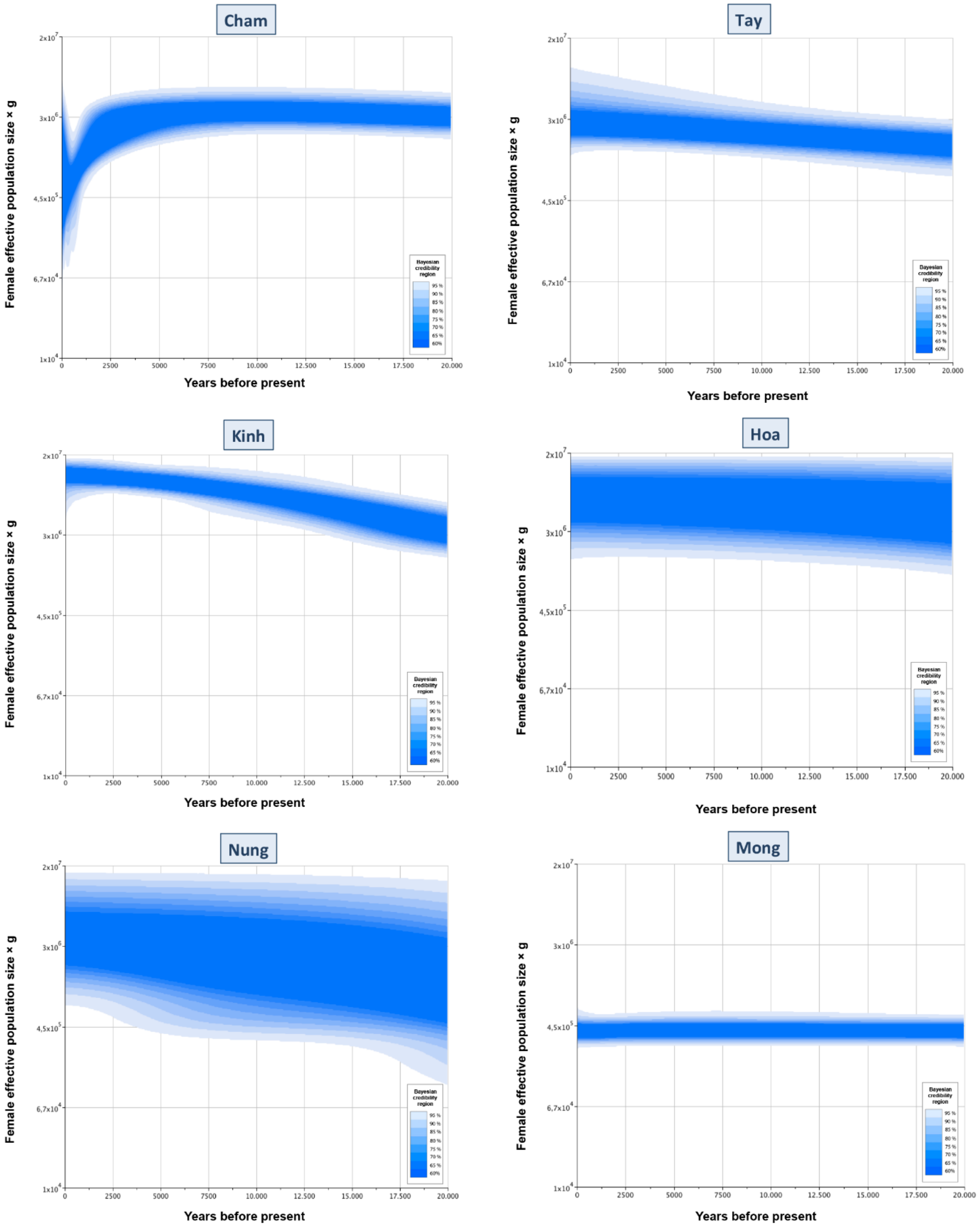
Cham
Tay
Kinh
Hoa
Nung
Mong
Female effective population size × g
Years before present
2x10^7
3x10^6
4,5x10^5
6,7x10^4
1x10^4
0
2500
5000
7500
10.000
12.500
15.000
17.500
20.000
Bayesian credibility region
95 %
90 %
85 %
80 %
75 %
70 %
65 %
60%

Figure S5.

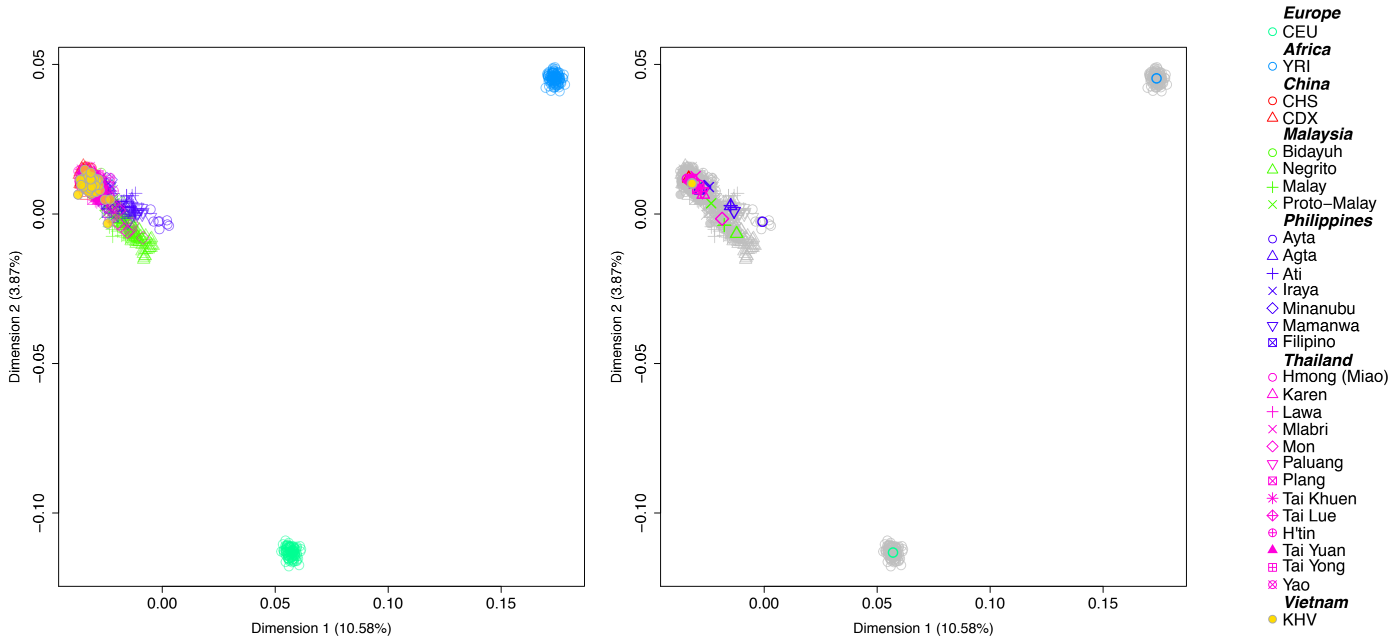
Dimension 1 (10.58%)
Dimension 2 (3.87%)
Europe
CEU
Africa
YRI
China
CHS
CDX
Malaysia
Bidayuh
Negrito
Malay
Proto–Malay
Philippines
Ayta
Agta
Ati
Iraya
Minanubu
Mamanwa
Filipino
Thailand
Hmong (Miao)
Karen
Lawa
Mlabri
Mon
Paluang
Plang
Tai Khuen
Tai Lue
H'tin
Tai Yuan
Tai Yong
Yao
Vietnam
KHV
